# Preparation of Functionalized Ethylene–Vinyl-Alcohol Nanofibrous Membrane Filter for Rapid and Cyclic Removing of Organic Dye from Aqueous Solution

**DOI:** 10.3390/polym16162328

**Published:** 2024-08-17

**Authors:** Jiachen Ding, Tingting Li, Xiangyi Wang, Mengyang Li, Tianyu Li, Zhiming Zhang

**Affiliations:** College of Material Science and Engineering, North China University of Science and Technology, Tangshan 063009, China; djc18732620983@163.com (J.D.); litingting2046@163.com (T.L.); 15732839308@163.com (X.W.); lmy65459@163.com (M.L.); 18713238678@163.com (T.L.)

**Keywords:** bifunctional nanofibrous filter, electrospinning, functionalized EVOH, selective dye adsorption, photocatalytic degradation

## Abstract

A functionalized ethylene–vinyl-alcohol (EVOH) nanofibrous membrane (NFM) was fabricated via co-electrospinning H_4_SiW_12_O_40_ (SiW_12_) and EVOH first, and then grafting citric acid (CCA) on the electrospun SiW_12_@EVOH NFM. Characterization with FT-IR, EDX, and XPS confirmed that CCA was introduced to the surface of SiW_12_@EVOH NFM and the Keggin structure of SiW_12_ was maintained well in the composite fibers. Due to a number of carboxyl groups introduced by CCA, the as-prepared SiW_12_@EVOH-CCA NFM can form a high number of hydrogen bonds with CR, and thus can be used to selectively absorb congo red (CR) in aqueous solutions. More importantly, the CR enriched in the NFM can be rapidly degraded via photocatalysis. SiW_12_ in the NFM acted as a photocatalyst, and the hydroxyl groups in the NFM acted as an electron donor to accelerate the photodegradation rate of CR. Meanwhile, the SiW_12_@EVOH-CCA NFM was regenerated and then exhibited a relatively stable adsorption capacity in five cycles of filtration–regeneration. The bifunctional nanofibrous membrane filter showed potential for use in the thorough purification of dye wastewater.

## 1. Introduction

Dye-containing wastewater has attracted increasing attention due to its intense color, high toxicity, and poor degradability [1,2]. The discharge of about one billion tons of dye wastewater per year has had a serious impact on the ecological environment and human health [3]; therefore, dye wastewater must be adequately treated before its release into natural water resources [4]. Currently, many techniques have been developed for the removal of dyes [5,6,7,8,9,10]; however, most of the organic dyes with complex aromatic molecular structures are highly stable to light and oxidation, so each technique has some limitations [11]. Recently, adsorption has been generally used in dye wastewater treatment owing to its low cost, high efficiency, easy operation, and no secondary pollution [12,13,14]. Accordingly, substantial efforts have been made to develop effective adsorbents, especially adsorbents with selective adsorption capacity for specific dyes [15,16,17].

Selective adsorption can achieve the effective separation of different dyes, which is beneficial for dye recycling. Li et al. [18] fabricated a pH-responsive adsorbent for the selective adsorption of dyes; when the solution was alkaline, only methylene blue (MB) could be adsorbed. In contrast, anionic dyes have been adsorbed in acidic solutions. Akbari et al. [19] employed TiO_2_ as matrix and encapsulated vanadium phosphorus oxide in it, and the obtained adsorbent exhibited high selective adsorption toward MB in the mixed-dye solutions; however, the majority of the used adsorbents were powdered materials, and reports on membrane adsorbents are still relatively few. Indeed, membrane adsorbents are more predominant compared to powdered adsorbents because no complex separation operations are required for the membrane during the recycling process.

Nanofibrous membranes (NFMs), fabricated by electrospinning, have greater surface areas than thin films [20]. In addition, electrospun NFMs have many unique properties, including high porosity, fine diameters, and ease of functionalization [21,22]. Our group has fabricated a metal–organic framework (MOF) NFM through the co-electrospinning of bio-MOF-1 and polyacrylonitrile (PAN). The as-prepared bio-MOF/PAN NFM can separate MB rapidly from mixed dyes. The selective adsorption mechanism of the MOF NFM toward MB was attributed to ion exchange, while chemical precipitation, electrostatic attraction, and hydrogen bonds also played an important role in the adsorption selectivity of different systems [23].

**Scheme 1:**
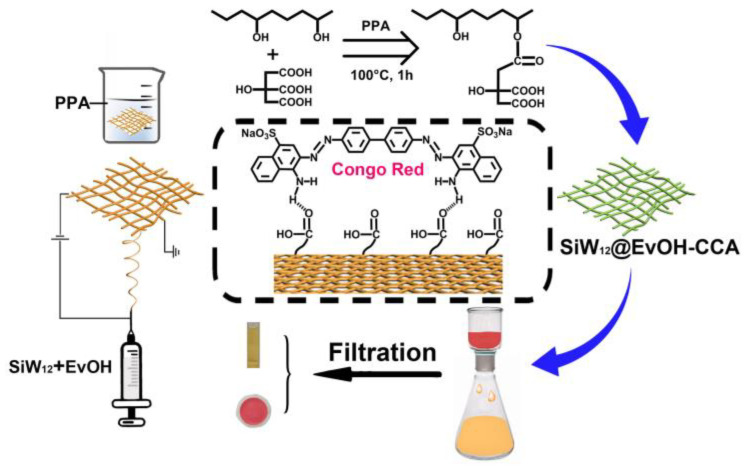
Preparation of SiW_12_@EVOH-CCA NFM and its selective adsorption of CR.

In this work, a NFM filter was prepared by grafting citric acid (CCA) onto an electrospun ethylene–vinyl-alcohol (EVOH) NFM for dye separation (Scheme 1). EVOH was chosen as a substrate primarily because of its good hydrophilicity, robust mechanical strength, and water insolubility [24]. Moreover, the hydroxyl groups in EVOH not only serve as the active sites for the dyes’ adsorption but also can be used for further derivatization [25]. CCA could introduce additional adsorption sites (carboxyl groups) to the surface of EVOH NFM, thereby EVOH-CCA NFM has a much better adsorption capacity. Based on the hydrogen bond interactions, the as-appeared EVOH-CCA filter can selectively and rapidly adsorb the congo red (CR) from aqueous solution. Moreover, as an azo dye, CR can be photocatalytically degraded by a variety of photocatalysts, such as V_2_O_5_/CdS [26], CeO_2_ [27], and NiCo_2_S_4_/CS [28]. In view of this, when H_4_SiW_12_O_40_ (SiW_12_) was blended into EVOH NFM via co-electrospinning before grafting the CCA, the CR enriched in the NFM was degraded by photocatalysis, and meanwhile, the SiW_12_@EVOH-CCA NFM was recovered. This is the first report of a bifunctional nanofibrous membrane filter for selective separation and photocatalytic degradation of CR from aqueous solution.

## 2. Materials and Methods

### 2.1. Materials and Apparatus

EVOH (27 mol% ethylene content) was purchased from Kuraray Co., Ltd. (Tokyo, Japan). Polyphosphoric acid (PPA), CCA, isopropyl alcohol, and congo red (CR) were purchased from Macklin Biochemical Co., Ltd. (Shanghai, China). SiW_12_, methyl orange (MO), crystal violet (CV), and solvent yellow 2 (SY2) were purchased from Aladdin Reagent Co., Ltd. (Shanghai, China). All chemicals and reagents were of analytical grade.

The SiW_12_@EVOH-CCA NFMs and their adsorption performances were mainly characterized by scanning electron microscopy (SEM, Hitachi S-4800, Tokyo, Japan), Fourier-transform infrared spectroscopy (FT-IR, VETERX70, Karlsruhe, Germany), X-ray photoelectron spectroscopy (XPS, ESCALAB 250Xi, London, Britain), BET analysis instrument (3H-2000PS1, Beishide, Beijing, China), and UV–vis spectrophotometer (8000S, Metash, Shanghai, China).

### 2.2. Fabrication of SiW_12_@EVOH NFM

EVOH (0.74 g) was dissolved in a mixture solvent (10 mL) of deionized water and isopropyl alcohol (3/7, *v*/*v*) with magnetic stirring (200 RPM) at 55 °C (water bath heating). After cooling to room temperature, 0.2 g SiW_12_ was dissolved in the above solution. The obtained electrospinning solution was injected into a glass syringe (5 mL). In a typical experimental procedure, the used voltage was 17 kV, the collection distance was 15 cm, and the flow rate of the solution was 0.4 mL h^−1^.

### 2.3. Preparation of SiW_12_@EVOH-CCA NFM

PPA (0.1 g) and CCA (0.9 g) were dissolved in deionized water (15 mL). Then, the SiW_12_@EVOH NFM was immersed in the solution for 1 h. The soaked SiW_12_@EVOH NFM was tiled on a PTFE plate and placed in a blast-drying oven. The reaction temperatures were set at 80 °C, 90 °C, and 100 °C, respectively. One hour later, the SiW_12_@EVOH-CCA NFMs—modified at different temperatures—were obtained and washed with deionized water.

### 2.4. Selective Adsorption of CR by SiW_12_@EVOH-CCA NFM

The trimmed SiW_12_@EVOH-CCA NFM with a diameter of 5.4 cm was fixed on a filter. The adsorption of dyes was performed by suction filtration (10 kPa). CR solution (30 mL, 100 mg L^−1^) was dumped onto the filter and forced to flow through the SiW_12_@EVOH-CCA NFM five times successively. The UV–vis spectrophotometer was used to measure the concentration change in CR, and its characteristic absorption peak appeared at 506 nm. The evaluation of the selective separation performance of the SiW_12_@EVOH-CCA NFM was performed in a similar manner. CR/MO, CR/CV, and CR/SY2 mixed-dye solutions were selected as models. The adsorption capacity and removal efficiency of the dyes were calculated according to the following formulas:

(1) $$q=\frac{(C_{0}-C)V}{W}$$

(2) $$\mathrm{Removal}(\%)=\frac{\left(C_{0}-C\right)}{C_{0}}\times 100\%$$

where q (mg g^−1^) represents the adsorption capacity of the NFMs toward organic dyes; C_0_ and C (mg mL^−1^) are the concentrations of the dyes, initially and after each filtration, respectively; W (g) is the weight of the SiW_12_@EVOH-CCA NFM; and V (mL) is the volume of dyes solution.

### 2.5. Regeneration and Reuse of SiW_12_@EVOH-CCA NFM

The recovery process was to adopt the photocatalytic technology to degrade CR enriched in the SiW_12_@EVOH-CCA NFM. The NFM after adsorption was placed in deionized water (50 mL) and was exposed to a 300 W high-pressure mercury lamp, which was located at a distance of 10 cm above the solution. The solution was placed in a double-layer quartz container, and cooled by condensate water during the experiment to ensure that the photocatalytic degradation was conducted at room temperature. At given intervals of illumination (1 h), the NFM was taken out for photographs. The photocatalytic reaction lasted for several hours until the color of the SiW_12_@EVOH-CCA NFM changed from red to white. The recovered NFM was continued for the next adsorption experiment under the previous conditions, and the filtration–regeneration process was performed in 5 consecutive cycles.

## 3. Results and Discussion

### 3.1. Characterization of SiW_12_@EVOH-CCA NFM

**Figure 1:**
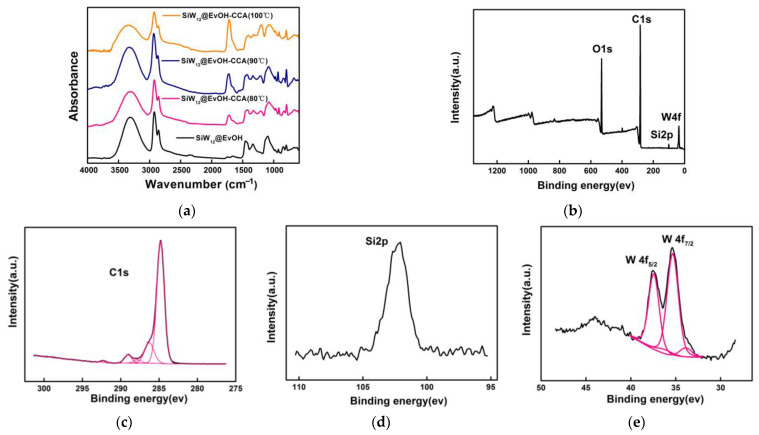
(**a**) FT-IR spectra of SiW_12_@EVOH-CCA NFMs produced at different grafting temperatures. (**b**) XPS survey spectrum of SiW_12_@EVOH-CCA. (**c**) C 1s scan, (**d**) Si 2p scan, and (**e**) W 4f scan.

The FT-IR spectra of SiW_12_@EVOH-CCA NFMs, produced with different grafting temperatures, are displayed in Figure 1a. For SiW_12_@EVOH NFM, a broad peak around 3396–3224 cm^−1^ was assigned to the vibrations of hydroxyl groups in EVOH. Three peaks at 971 cm^−1^, 917 cm^−1^, and 794 cm^−1^ were attributed to the ν_as_(W=O_d_), ν_as_(W-O_b_-W), and ν_as_(W-O_c_-W), respectively [29]. After modification with CCA, a peak at 1716 cm^−1^ appeared, which represented the stretching vibrations of C=O [30]. It is worth noting that the intensity of this peak increased as the grafting temperature increased from 80 °C to 100 °C, suggesting that high temperature can lead to an increased grafting rate. Figure 1b–e show the XPS measurement of SiW_12_@EVOH-CCA NFM. There were four spectral components with binding energies of 284.8, 286.3, 287.8, and 289.0 eV that appeared in the C 1s spectrum, attributed to C-C, C-O, C=O, and O-C=O, respectively [31]. The O-C=O occupied 4.12% of the spectral area, implying that the SiW_12_@EVOH-CCA NFM had sufficient carboxyl groups on its surface. From Figure 1e, the spectrum of W 4f was deconvoluted into two components. Peaks at 35.4 and 37.5 eV were assigned to W 4f_7/2_ and W 4f_5/2_, supporting the existence of W (VI). W (VI) occupied a large proportion of the spectral area, and this valence state would play a major role in the photocatalytic reaction.

**Figure 2:**
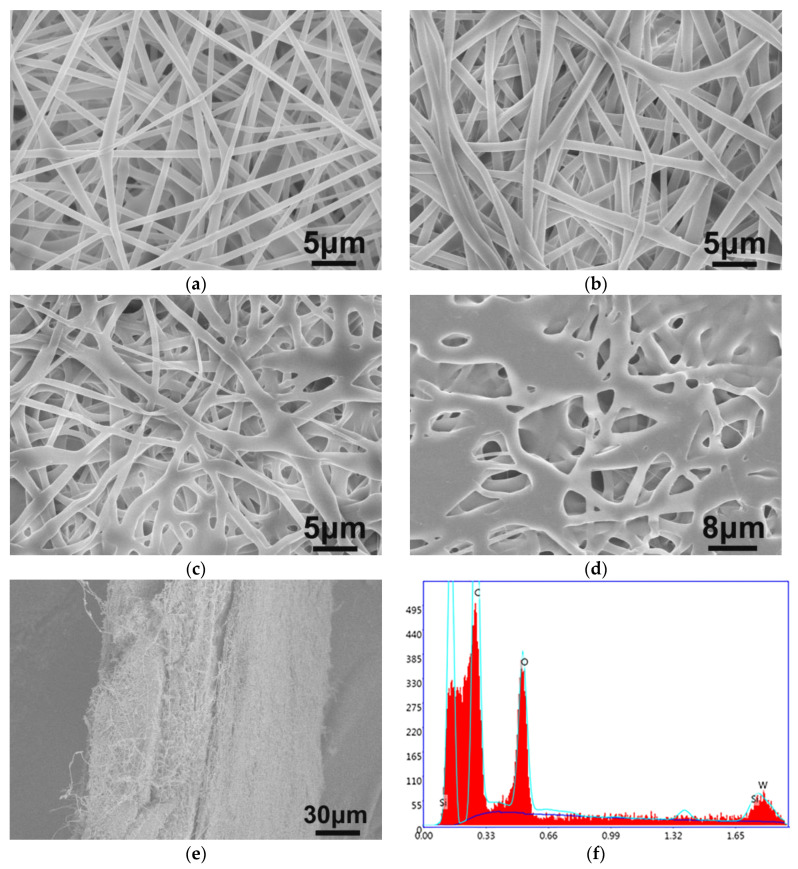
(**a**) SEM images of SiW_12_@EVOH nanofibers. SEM images of SiW_12_@EVOH-CCA NFMs produced at different grafting temperatures: (**b**) 80 °C, (**c**) 90 °C, (**d**) 100 °C. Cross-sectional SEM (**e**) and EDX (**f**) for SiW_12_@EVOH-CCA NFM.

**Figure 3:**
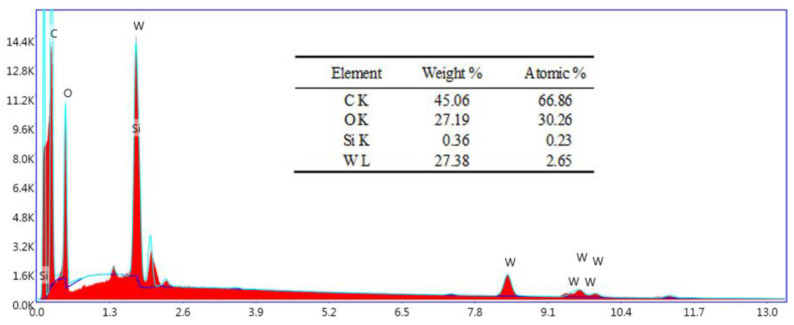
EDX spectrum of SiW_12_@EVOH-CCA NFM with grafting temperature of 90 °C.

Figure 2a displays the representative morphology of the SiW_12_@EVOH fibers. The random fibers present a smooth surface with a uniform diameter. After modification by CCA, these fibers showed different changes at different grafting temperatures. From Figure 2b, when the grafting temperature was 80 °C, the fibers’ morphology was similar to the original one. The fibers began to adjoin to each other as the grafting temperature rose to 90 °C, as shown in Figure 2c. At this temperature, SiW_12_@EVOH-CCA NFM can still basically maintain its fiber structure. However, the adhesion between nanofibers became serious at 100 °C, and the fiber structure almost disappeared (Figure 2d). This compact structure reduces the porosity of SiW_12_@EVOH-CCA NFM, which is unfavorable for filtration. In view of this, 90 °C was selected as the grafting temperature for the subsequent experiment. The composition of the SiW_12_@EVOH-CCA NFM was examined by EDX, as shown in Figure 3. It was clear that the Si and W elements appeared in the spectrum, further indicating the existence of SiW_12_ in the NFM. Furthermore, the atomic ratio of Si and W was approximately 1:11.5 in the EDX analysis, which was very close to the atomic ratio of Si and W in SiW_12_.

**Figure 4:**
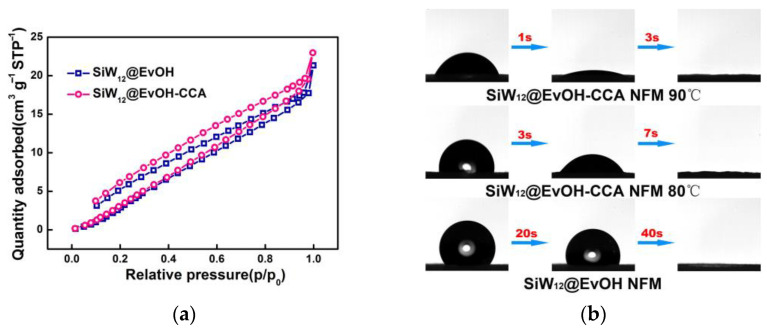
(**a**) N_2_ adsorption–desorption isotherms of SiW_12_@EVOH and SiW_12_@EVOH-CCA NFM. (**b**) Water contact angles of SiW_12_@EVOH and SiW_12_@EVOH-CCA NFM.

The N_2_ adsorption–desorption isotherm of SiW_12_@EVOH-CCA NFM is shown in Figure 4a. The isotherm curve showed a similar adsorption shape (IV type) to the SiW_12_@EVOH NFM, which indicated the existence of macropores in the NFMs. A slight decrease (from 48.1 to 42.2 m^2^ g^−1^) in the BET surface area occurred after grafting CCA may be due to the adhesion structure of the nanofibers after the grafting reaction. Figure 4b displays the wettability of SiW_12_@EVOH-CCA NFM. The initial water contact angle (WCA) of SiW_12_@EVOH NFM was 127.8° and the water droplet was infiltrated completely after 40 s. When the grafting temperature was 80 °C, SiW_12_@EVOH-CCA NFM exhibited an initial WCA of 98.0° and its complete infiltration time was reduced to 7 s. The hydrophilicity of SiW_12_@EVOH-CCA NFM further increased as the grafting temperature was up to 90 °C. This phenomenon was mainly because CCA can introduce carboxyl groups to SiW_12_@EVOH NFM. As the main adsorption sites, the content of carboxyl groups increased with the grafting temperature. Both a large specific surface area and good hydrophilicity facilitated full contact of the dyes and adsorption sites, leading to a rapid and sufficient adsorption effect.

### 3.2. Selective Adsorption of Dyes by SiW_12_@EVOH-CCA NFM

**Figure 5:**
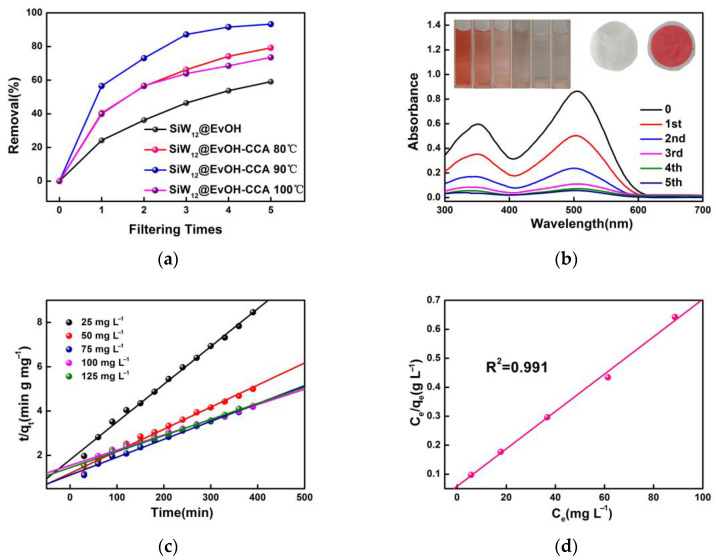
(**a**) Removal efficiency of CR by SiW_12_@EVOH-CCA NFM filters produced at different grafting temperatures. (**b**) UV–vis spectra of CR in the filtration process. Pseudo-second-order kinetic model (**c**) and Langmuir adsorption isotherm (**d**) of SiW_12_@EVOH-CCA NFM toward CR.

Based on the hydrogen bond interactions, SiW_12_@EVOH containing hydroxyl groups exhibited a 59.10% removal of CR, as shown in Figure 5a. After grafting CCA, the removal efficiency of CR by the modified NFMs increased significantly. A total of 79.17%, 93.30%, and 73.51% removal efficiency was observed by the SiW_12_@EVOH-CCA NFMs with grafting temperatures of 80 °C, 90 °C, and 100 °C, respectively. This was mainly because CCA introduced a certain amount of -COOH to the SiW_12_@EVOH NFM, which can improve the binding ability toward CR. The removal efficiency of the NFM grafted at 100 °C was less than that of the NFM grafted at 90 °C, which may be attributed to its compact fiber structure. Figure 5b shows the UV–vis spectra of CR after five successive filtrations. The absorption peak of CR decreased with the increase in filtration times. Meanwhile, the color of CR gradually changed from red to colorless, and the SiW_12_@EVOH-CCA NFM changed from white to red. The water flux of this NFM was 2304 L m^−2^ h^−1^. All the results indicate that the SiW_12_@EVOH-CCA NFM had an excellent adsorption capacity for CR.

The adsorption kinetics were investigated as follows. A total of 40 mg SiW_12_@EVOH-CCA NFM was immersed in CR solutions (100 mL) with different initial concentrations, and then the solutions were shaken until the adsorption reached equilibrium. From Figure 5c, the removal processes of CR by SiW_12_@EVOH-CCA NFM followed well with the pseudo-second-order kinetic model [32].

(3) $$\frac{t}{\mathrm{qt}}=\frac{1}{{\mathrm{kq}}_{e}^{2}}+\frac{t}{\mathrm{qe}}$$

where *q_t_* and *q_e_* are the adsorption capacity at time *t* and equilibrium (mg g^−1^), respectively; *k* is the adsorption rate constant of pseudo-second-order. The correlation coefficients (R^2^) for the different initial concentrations of CR were above 0.99, suggesting that the removal of CR by SiW_12_@EVOH-CCA NFM was mainly through chemisorption [33]. Figure 5d displays the Langmuir adsorption isotherm of SiW_12_@EVOH-CCA NFM toward CR. It was clear that R^2^ reached 0.991, demonstrating that the Langmuir model [32] was very suitable for the analysis of this experimental data.

(4) $$\frac{C_{e}}{q_{e}}=\frac{C_{e}}{q_{\max}}+\frac{1}{{\mathrm{KL}}_{\mathrm{qmax}}}$$

where *C_e_* (mg L^−1^) is the equilibrium concentration of CR; *q_e_* and *q_max_* are the adsorption capacity at equilibrium and the maximum adsorption capacity (mg g^−1^), respectively; *K_L_* is the Langmuir constant. The morphology of this relevant fitted curve illustrates that the adsorption of CR by the NFM obeyed monolayer adsorption.

In addition, the CR initial concentration and filtration method were also studied as parameters that may affect the adsorption effect. When the initial concentrations of CR were 25 mg L^−1^ and 50 mg L^−1^, the removal efficiency reached more than 98% within less than five filtrations. The removal efficiency was 96.17%, 93.30%, and 73.10% when the initial concentration of CR was 75 mg L^−1^, 100 mg L^−1^, and 125 mg L^−1^, respectively. When the initial concentration of CR was higher than 100 mg L^−1^, the removal efficiency began decreasing obviously. In terms of the filtration method, suction filtration and gravity filtration have been compared. Under conditions where all the other parameters were the same, one round of suction filtration took about 20 s, while one round of gravity filtration took about 45 min; however, the difference in the removal efficiency between the two filtration methods was only 9.18%. Therefore, suction filtration was used in the next experimental operation.

**Figure 6:**
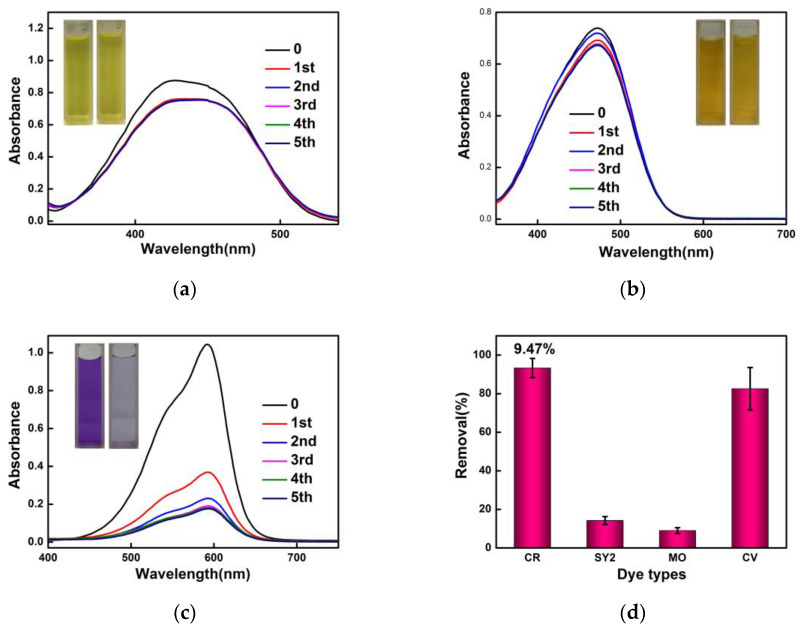
UV–vis spectra of (**a**) SY2, (**b**) MO, and (**c**) CV in the filtration process. The insets were the dye solutions before and after filtration. (**d**) The removal efficiency of SY2, MO, and CV.

Three other dyes (MO, SY2, and CV) were employed to evaluate the selective separation ability of SiW_12_@EVOH-CCA NFM. From Figure 6a,b, it can be seen that the absorption peaks of SY2 and MO declined slightly, and their solution color showed very little change. This was mainly because there were no functional groups in the structures of SY2 and MO that could interact with the SiW_12_@EVOH-CCA NFM. In contrast, the characteristic peaks of CV decreased significantly when the filtration time was increased. At the same time, the color of the CV solution gradually faded (Figure 6c). The adsorption of CV by SiW_12_@EVOH-CCA NFM was mainly attributed to the electrostatic attraction. The membrane surface contained a lot of carboxyl groups, while CV was a cationic dye. As a result, the removal efficiency of SY2, MO, and CV were 14.22%, 9.02%, and 82.59%, respectively (Figure 6d).

**Figure 7:**
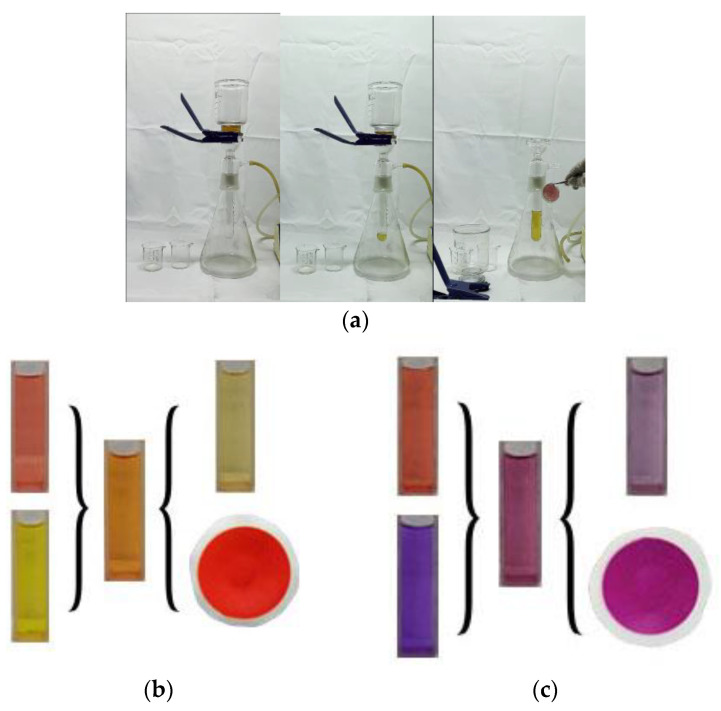
The color change in the mixed dye solutions and SiW_12_@EVOH-CCA NFM before and after filtration: (**a**) CR/MO, (**b**) CR/SY2, and (**c**) CR/CV.

**Figure 8:**
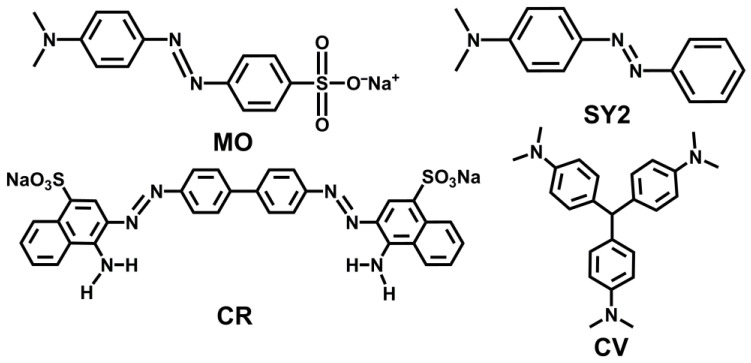
Structures of the four dyes.

The separation of mixtures of CR/MO, CR/SY2, and CR/CV were carried out to further investigate the selective adsorption capacity of the NFM. As shown in Figure 7a and Supplementary Video S1, after about 20 s of suction filtration, the orange–red solution of CR mixed with MO turned yellow, which was the color of the MO solution. Meanwhile, the SiW_12_@EVOH-CCA NFM exhibited a red color. This phenomenon suggested that only CR can be absorbed by SiW_12_@EVOH-CCA NFM in the mixture solution. The amino groups in CR can undergo hydrogen bond interactions with carboxyl groups in SiW_12_@EVOH-CCA NFM, while MO has no groups that can form effective hydrogen bonds. A similar separation effect also occurred in a mixed solution of CR/SY2, as shown in Figure 7b. However, for CR/CV (Figure 7c), the adsorption of CR and CV occurred successively. CR was preferentially adsorbed based on hydrogen bonds, and then CV was removed via electrostatic attraction. Eventually, the solution turned light purple, while the NFM changed from white to purplish-red. The structures of the above four dyes are displayed in Figure 8.

### 3.3. Recycling Process of SiW_12_@EVOH-CCA NFM

**Figure 9:**
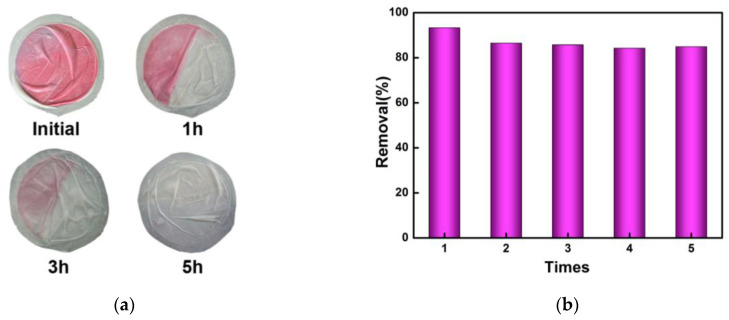
(**a**) The images of the SiW_12_@EVOH-CCA NFM after photocatalytic degradation. (**b**) The removal efficiency of CR by SiW_12_@EVOH-CCA NFM after 5 cycles.

The reusability of the separation membrane was very important for long-term use [34]. The SiW_12_@EVOH-CCA NFM can be recovered by photocatalytic degradation of the adsorbed CR. SiW_12_ in the NFM acted as a photocatalyst for CR degradation. It was reported that in the presence of alcohol as a sacrificial electron donor, the photodegradation process can be driven by the reductive pathway with a fast degradation rate [35,36]. Our previous work demonstrated that -OH can also donate an electron to SiW_12_ [37]. In view of this, the hydroxyl groups in SiW_12_@EVOH-CCA NFM acted as an electron donor to accelerate the photodegradation rate of CR, and the degradation effect is displayed in Figure 9a. As the photocatalytic time increased, the red color of the NFM gradually became lighter. After 5 h of catalysis, the SiW_12_@EVOH-CCA NFM basically returned to white, indicating that the CR adsorbed in the NFM had been almost completely degraded. The recovered SiW_12_@EVOH-CCA NFM was continued for the next adsorption, and the removal efficiency of CR was 84.90% after five cycles, which was just 8.9% less than that of the first filtration.

## 4. Conclusions

A functionalized EVOH NFM was prepared by co-electrospinning SiW_12_ and EVOH first, and then grafting CCA on the electrospun SiW_12_@EVOH NFM. The obtained SiW_12_@EVOH-CCA NFM has been used as a filter to remove CR from aqueous solution. Benefiting from a number of carboxyl groups introduced by CCA, the functionalized NFM can form a high number of hydrogen bonds with CR, resulting in rapid adsorption and selective separation of CR from mixed-dye solution. After about 20 s of suction filtration, CR can be successfully separated from mixed dyes. More importantly, the SiW_12_@EVOH-CCA NFM can be effectively regenerated via photocatalytic degradation. SiW_12_ in the NFM acted as a photocatalyst, and the hydroxyl groups in the NFM acted as an electron donor to accelerate the photodegradation rate of CR. The removal efficiency of CR using the recovered SiW_12_@EVOH-CCA NFM had only an 8.9% decrease after five filtration–regeneration cycle experiments. This study provides a new perspective on the preparation of bifunctional electrospun NFMs, which can be applied to the thorough purification of practical dye wastewater.

## Data Availability

The original contributions presented in the study are included in the article, further inquiries can be directed to the corresponding author.

## Supplementary Materials

The following supporting information can be downloaded at: https://www.mdpi.com/article/10.3390/polym16162328/s1, Video S1: The separation of mixtures of CR/MO.
